# A new inducible model for t(8;21) AML

**DOI:** 10.1002/emmm.201303483

**Published:** 2013-11-04

**Authors:** James C Mulloy

**Affiliations:** Division of Experimental Hematology and Cancer Biology, Cincinnati Children's Hospital Medical Center, University of Cincinnati College of MedicineCincinnati, OH, USA

**Keywords:** acute myeloid leukaemia, AML1-ETO, inducible mouse-model, t(8;21)

Faithful and tractable mouse models for t(8;21)-associated human acute myeloid leukaemia (AML) have been difficult to develop, limiting insight into the malignancy associated with this frequent chromosomal translocation. In this issue of *EMBO Molecular Medicine*, Cabezas-Wallscheid et al. bring us a step closer to this goal with an improved Tet-inducible mouse model of t(8;21)-associated AML (Cabezas-Wallscheid et al, 2013).

The t(8;21) is found in approximately 10% of human AML and encodes for the leukaemia fusion protein AML1-ETO (RUNX1-RUNX1T1) (Miyoshi et al, 1993). The translocation results in a fusion protein comprised of the amino-terminal part of RUNX1, including the conserved RUNT domain that is critical for function, and nearly the entire coding region of the ETO (RUNX1T1) protein. RUNX1 forms a heterodimeric transcription factor complex with the CBFβ protein. This complex is known as core binding factor (CBF), and both the RUNX1 gene on chromosome 21 and the CBFβ gene on chromosome 16 are involved in chromosomal translocations in 15–20% of human AML. CBF is a master regulator of haematopoiesis and knockout of either RUNX1 or CBFβ results in embryonic lethality due to failure of definitive haematopoiesis (Chuang et al, 2013). The ETO protein is a transcriptional repressor that associates with nuclear co-repressor proteins and histone deacetylase activity. Although the molecular mechanisms through which AML1-ETO exerts its effects are unclear, it is likely that the strong repressor function contributed by ETO is critical. Repression of numerous genes involved in haematopoietic differentiation, including CEBPA and SPI1 (PU.1), likely plays an important role in the functional outcome upon expression of AML1-ETO. However, it is clear from various studies that AML1-ETO is not solely functioning as a strong transcriptional repressor of CBF activity. Many genes are directly up-regulated by AML1-ETO, and the molecular mechanisms involved in these processes are the subject of efforts in many laboratories around the globe. Dissecting the effects of AML1-ETO on haematopoietic differentiation and self-renewal and determining how these effects contribute to the final malignant phenotype remain a goal of leukaemia researchers (Lam and Zhang, 2012).

t(8;21) AML is generally associated with a good prognosis, with 60–70% of patients being cured of their disease in response to standard chemotherapy. Nevertheless, 30–40% of t(8;21) patients relapse and the prognosis for relapsed AML is dismal (Ofran and Rowe, 2012). A better understanding of the molecular mechanisms whereby AML1-ETO exerts its effects could reveal new therapeutic targets. Numerous approaches have been used to model this disease over the two decades since the identification of the t(8;21), with varying success and no accepted consensus as to which approach is both faithful to the human disease and relatively tractable as a model system (Hatlen et al, 2012). In this issue of *EMBO Molecular Medicine*, Cabezas-Wallscheid et al describe a new inducible model of t(8;21) malignancy that appears to satisfy these conditions and will undoubtedly improve our understanding of AML1-ETO function (Cabezas-Wallscheid et al, 2013). They employ a Tet-On inducible mouse model in which the rtTA is driven from the endogenous ROSA26 locus and leads to mosaic expression in the various blood cell lineages, including the haematopoietic stem cell (HSC; Wörtge et al, 2010). Using a chimera approach so as to focus on blood cells, they show expression of AML1-ETO in all analysed blood cell lineages at frequencies ranging from 3 to 10%, using EGFP as a surrogate marker for AML1-ETO expression. In isolated cells, a broad range of expression of the AML1-ETO transcript is detected in the different blood cell types, with highest expression in granulocyte–macrophage progenitors (GMP), at three- to fourfold higher expression levels relative to those detected in the patient-derived t(8;21) cell line Kasumi-1. Interestingly, as with most other models of AML1-ETO disease, no phenotype is evident during the first few months after induction of AML1-ETO. However, beginning at 9 months post-induction, the mice begin to develop aspects of what can be characterized as a pre-leukaemia, with anaemia, increased myelopoiesis, decreased lymphopoiesis and extramedullary haematopoiesis. This condition progresses over the next 6–8 months and results in a myeloproliferative disease (MPD)-like myeloid leukaemia, with increased blasts in the peripheral blood, bone marrow and spleen, and widespread infiltration of blasts into numerous organs. The disease is fully penetrant, transplantable, and expression studies demonstrate that the malignant cells show transcriptional changes that closely mimic signals associated with AML1-ETO disease in humans. This model opens multiple avenues of investigation that could lead to a better understanding of the cellular and molecular effects of AML1-ETO expression and the changes that occur as cells evolve during disease progression.

Given the inducible nature of this model, it was natural to ask whether cells acquire a dependence on AML1-ETO expression or whether disease continues and progresses even upon termination of this signal. Unfortunately the answer to this question remains unclear and more work is required to determine whether the initiating oncogene is still required during the terminal stages of leukaemia evolution. This is due to the fact that while nearly 50% of the mice regressed upon termination of the AML1-ETO signal (upon doxycycline removal), over 50% of the mice showed disease progression. Whether this results from cooperating events that substitute for the AML1-ETO signal or is due to incomplete loss of AML1-ETO expression upon doxycycline removal remains to be determined. This is an important question, given the efforts that are focused on targeting the AML1-ETO protein directly (Cunningham et al, 2012).

The nature of the cell associated with disease initiation and maintenance is a topic of great interest in leukaemia research (Konopleva and Jordan, 2011). In this Tet-inducible model, the primary cell that showed numeric expansion was the GMP, while other stem and progenitor subsets were either not affected or were diminished in number upon AML1-ETO expression. Interestingly, at the MPD-like myeloid leukaemia stage, both the GMP and HSC are able to transfer disease to secondary recipients, demonstrating that the leukaemic GMP (L-GMP) has acquired self-renewal abilities. The association of GMP-like cells with leukaemia disease propagation has been shown in a number of different models, but it remains an open question whether the expansion of the GMP population and the subsequent ability to transfer disease with a L-GMP are causally linked. It will be of interest to analyse other cellular compartments that do not show AML1-ETO-induced expansion, including the short term-HSC, MPP and CMP, and determine their leukaemia-initiating capacity, both during disease manifestation and long before disease is apparent. Is the expansion of the GMP due to an enhanced block of differentiation at this particular stage rather than an indication that this is the cell type that is particularly affected by AML1-ETO expression? Does expression of AML1-ETO immediately confer such abilities to stem and progenitor cells, in the absence of apparent disease? These are just a few of the questions that can now be addressed using this model.

Numerous approaches have been used to model this disease over the two decades since the identification of the t(8;21), with varying success and no accepted consensus as to which approach is both faithful to the human disease and relatively tractable as a model system…

The authors performed a number of different gene expression comparisons to determine the nature of the signals associated with short-term expression of AML1-ETO as well as the signals present at the time of disease manifestation in the L-GMP. In essence, attempting to identify AML1-ETO-specific signals as well as cooperating signals important for disease progression. The new model faithfully recapitulated many of the findings that have been made in other model systems, and showed a gene expression pattern that mirrors the signature found in t(8;21) blast samples expressing AML1-ETO. In addition, the nature of the cooperating events in the L-GMP, identified by comparing signatures from Day 10 AML1-ETO-induced GMP and L-GMP from overtly leukaemic animals, fit with patterns expected from numerous studies, implicating cellular signalling pathways associated with transformation, survival and proliferation as critical for cooperation with AML1-ETO signalling. Although this particular study utilized the GMP and L-GMP in comparative analyses, it will be of great interest to repeat these studies using HSC in place of GMP. While many human AML samples do in fact have populations with cellular phenotypes resembling progenitor cells (L-GMP-like, such as CD34+CD38+) that demonstrate leukaemogenic activity in xenograft studies, the vast majority of human AML patient samples show highest leukaemic activity in cells that resemble HSC (CD34+CD38−) (Bonnet and Dick, 1997; Goardon et al, 2011; Sarry et al, 2011). This may indicate that leukaemia initiation typically occurs in a more primitive cellular compartment, with some less primitive cell types acquiring leukaemic activity with time. The signature associated with these more primitive cells may be more representative of the majority of human AML samples.

Many questions remain unanswered regarding the nature of the signals downstream of AML1-ETO and the particular cooperating events that lead to leukaemia progression in the context of this oncogene. This novel inducible model represents a new tool in the arsenal for leukaemia researchers. In addition, it can serve as a powerful therapeutic screening model once the appropriate cooperating events are supplied to allow progression to a lethal AML disease.

Conflict of interest statement: J.C.M. is a Scholar of the Leukemia and Lymphoma Society. The author declares that he has no conflict of interest.
